# Copper-Based Electrocatalysts for Nitrate Reduction to Ammonia

**DOI:** 10.3390/ma16114000

**Published:** 2023-05-26

**Authors:** Jia-Yi Fang, Jin-Long Fan, Sheng-Bo Liu, Sheng-Peng Sun, Yao-Yin Lou

**Affiliations:** 1 School of Chemical and Environmental Engineering, College of Chemistry, Chemical Engineering and Materials Science, Soochow University, Suzhou 215123, China; 2 State Key Laboratory of Physical Chemistry of Solid Surfaces, Department of Chemistry, College of Chemistry and Chemical Engineering, Xiamen University, Xiamen 361005, China; 3 Jiangsu Key Laboratory of Environmental Science and Engineering, School of Environmental Science and Engineering, Suzhou University of Science and Technology, Suzhou 215009, China

**Keywords:** nitrate reduction, nitrogen cycle, mechanism, ammonia

## Abstract

Ammonia (NH_3_) is a highly important industrial chemical used as fuel and fertilizer. The industrial synthesis of NH_3_ relies heavily on the Haber–Bosch route, which accounts for roughly 1.2% of global annual CO_2_ emissions. As an alternative route, the electrosynthesis of NH_3_ from nitrate anion (NO_3_^−^) reduction (NO_3_^−^RR) has drawn increasing attention, since NO_3_^−^RR from wastewater to produce NH_3_ can not only recycle waste into treasure but also alleviate the adverse effects of excessive NO_3_^−^ contamination in the environment. This review presents contemporary views on the state of the art in electrocatalytic NO_3_^−^ reduction over Cu-based nanostructured materials, discusses the merits of electrocatalytic performance, and summarizes current advances in the exploration of this technology using different strategies for nanostructured-material modification. The electrocatalytic mechanism of nitrate reduction is also reviewed here, especially with regard to copper-based catalysts.

## 1. Introduction

As an important industrial chemical, ammonia (NH_3_) is widely used in synthetic drugs, fertilizers, dyes, plastics, and so on [1,2]. NH_3_ is also regarded as a carbon-free and environmentally friendly clean-energy carrier of hydrogen, since NH_3_ is easier to liquefy, store, and transport than hydrogen [3,4]. To date, the industrial synthesis of NH_3_ has relied heavily on the Haber–Bosch route. This route requires harsh conditions of high temperature (400–600 °C) and high pressure (200–350 atm) [5]. In fact, the energy consumption of the Haber–Bosch process accounts for 1.4% of the world’s total annual energy consumption, and the resulting carbon dioxide emissions are as high as 450 million tons, accounting for approximately 1.2% of global annual carbon dioxide emissions [6]. Therefore, researchers began to look for low-carbon and environmentally friendly alternatives for producing NH_3_ that can be realized under normal temperature and pressure conditions. Among many alternatives, the method of producing NH_3_ from nitrogen (N_2_) and H_2_O driven by renewable electric energy (NRR) has gradually attracted the researchers’ attention [7,8,9]. However, due to the high bond energy (941 kJ mol^−1^) of the N≡N bond and the low solubility of N_2_ in water, the energy utilization of NRR in the aqueous environment is low, and its production yield of NH_3_ is 2–3 orders of magnitude lower than the Haber–Bosch process. As the “Renewable Energy to Fuels through Utilization of Energy-dense Liquids” (REFUEL) program of the Department of Energy (DOE) demonstrates, the electrochemical synthesis of NH_3_ requires the realization of an NH_3_-production Faraday efficiency exceeding 90%, an NH_3_ yield of 10^−6^ mol s^−1^ cm^−2^, a current density of 300 mA cm^−2^, and an energy efficiency of 60% for industrial production [10]. It is very difficult to achieve these indicators through the NRR process, which limits the further development of NRR [11]. For this reason, researchers have tried to apply other nitrogen-containing compounds as nitrogen sources to synthesize NH_3_ [12]. Nitrate anion (NO_3_^−^) is a good candidate, since the solubility of NO_3_^−^ in water is high and the bond energy of N=O bond (204 kJ mol^−1^) is low [13]. The NO_3_^−^ reduction process is a solid–liquid interface reaction, which is more conducive to mass transfer than a gas–liquid–solid interface reaction during NRR and has the potential to realize the target of the REFUEL program. NO_3_^−^ is a pollutant that widely exists in industrial and agricultural wastewater and domestic sewage [14]. The residual NO_3_^−^ in the environment will not only harm the water body ecosystem and lead to water eutrophication but will also pose a serious threat to human health [15]. Therefore, using NO_3_^−^ as a nitrogen source for NH_3_ synthesis is not only conducive to improving energy utilization efficiency and reducing greenhouse gas emissions; it can also alleviate the problem of NO_3_^−^ pollution in the environment.

In the natural environment, the conversion of NO_3_^−^ to NH_3_ can be biocatalyzed by a nitrate/nitrite reductase (such as *Shewanella oneidensis* cytochrome *c* nitrite reductase) under mild environmental conditions (a process known as bio-NRA) [16]. NH_3_ can then be concentrated by physicochemical methods such as ion exchange adsorption and struvite precipitation and used for further industrial applications [6]. However, the biocatalytic process of bio-NRA takes a long time to effect the conversion, and the efficiency of NO_3_^−^ removal is low. In addition, the complex structures of the bio-enzymes are difficult to synthesize artificially. Therefore, scientists began to simulate the function of microbial enzymes to design and develop artificially synthesized catalytic materials to satisfy the demands of industrial production [17]. Research on the electrocatalytic reduction of NO_3_^−^ (NO_3_^−^RR) to NH_3_ is also facing great challenges. NO_3_^−^RR involves the transfer of 8e^−^ and 9 protons, during which various byproducts such as nitrite (NO_2_^−^), nitrogen monoxide (NO), nitrogen (N_2_), and nitrous oxide (N_2_O) are produced as well [18]. Meanwhile, there is competition between the hydrogen evolution reaction (HER) and NO_3_^−^RR. With the increase in the overpotential, HER will be increasingly strengthened, which further reduces the energy utilization efficiency of NH_3_ formation. Therefore, it is of great significance to carry out in-depth research on the mechanism of electrocatalytic NO_3_^−^RR synthesis of NH_3_ to rationally guide the development and design of catalysts with high catalytic activity and high NH_3_ formation selectivity.

## 2. Reaction Mechanism of Electrocatalytic NO_3_^−^RR

**Figure 1:**
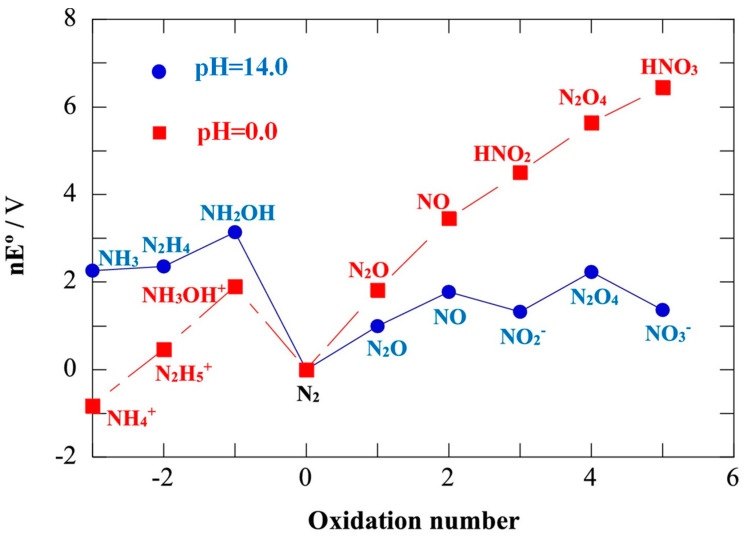
Frost–Ebsworth diagram [19].

N has a variety of stable forms of hydrides and oxides from −3 to +5 valence states. The thermodynamic relationship between these nitrides is shown in the Frost–Ebsworth diagram (Figure 1) [13]. A series of reaction intermediate species and final products would be formed during NO_3_^−^ reduction, and N_2_ and NH_3_ are the two most thermodynamically stable products, which can be obtained through the following Equations (1) and (2).

(1) $$2{NO}_{3}^{-}+12H^{+}+{10e}^{-}\to N_{2}+6H_{2}O\;E^{0}=1.17\;V\;vs.\;SHE$$

(2) $${NO}_{3}^{-}+9H^{+}+{8e}^{-}\to {NH}_{3}+3H_{2}O\;E^{0}=-0.12\;V\;vs.\;SHE$$

**Figure 2:**
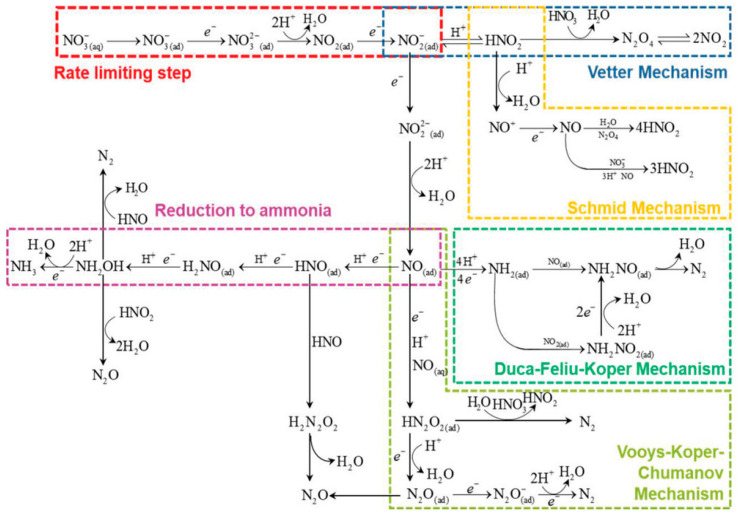
Schematic diagram of the mechanism of NO_3_^−^RR in aquatic media [13].

According to the literature, NO_3_^−^ reduction to NO_2_^−^ is the rate-determining step of NO_3_^−^RR (Figure 2) [20]. NO_3_^−^ in the solution is first adsorbed onto the cathode surface (Equation (3)), during which some disturbing anions in the solution may compete with NO_3_^−^ adsorption and inhibit NO_3_^−^ reduction [21]. The mass transfer rate of NO_3_^−^ from the solution to the electrode surface will also affect the adsorption process [22,23]. On the other hand, the number of active sites on the electrode’s surface will affect the reaction rate [23]. Currently, researchers propose two reaction mechanisms for NO_3_^−^RR to NH_3_: a direct reduction mechanism and an indirect autocatalytic reduction mechanism [19,24].

### 2.1. Direct Reduction Pathway

In the direct reduction pathway, the adsorbed NO_3_^−^ can be reduced by proton-coupling electrons or by adsorbed H atoms (*H) [19]. As shown in Equations (4)–(6), in the proton-coupling electron reduction pathway, the process of NO_3_^−^ being reduced to NO_2_^−^ involves three steps: (a) electrochemical (Equation (4)), (b) chemical (Equation (5)), and (c) electrochemical process (Equation (6)) [25,26,27]. Koper et al. [20] found that the Tafel slope of NO_3_^−^RR over transition metals in acidic solution is approximately 120 mV dec^−1^, and then they speculated that the first electron-transfer step is the rate-determining step. The slow kinetics of this step was attributed to the high energy of the lowest unoccupied π* orbital (LUMO π*) of NO_3_^−^, which made it difficult for electron transfer from the electrode surface into the LUMO π* of *NO_3_^−^ [28]. Cu with highly occupied d orbitals has a closed energy level with LUMO π* of NO_3_^−^. The electrons are easily transferred from the unclosed d orbital of Cu to the adsorbed NO_3_^−^, which is conducive to the electrochemical reduction of NO_3_^−^.

NO_3_^−^ adsorption:

(3) $${{NO}_{3}^{-}}_{aq}+*\to {{NO}_{3}^{-}}_{ad}$$

NO_3_^−^→NO_2_^−^:

(4) $${{NO}_{3}^{-}}_{ad}+e^{-}\to {{NO}_{3}^{2-}}_{ad}\;E^{0}=-0.89\;V\;vs.\;SHE$$

(5) $${{NO}_{3}^{2-}}_{ad}+H_{2}O\to {{NO}_{2}}_{ad}+2{OH}^{-}\;k=5.5\times 10^{4}s^{-1}$$

(6) $${{NO}_{2}}_{ad}+e^{-}\to {{NO}_{2}^{-}}_{ad}\;E^{0}=1.04\;V\;vs.\;SHE$$

NO_2_^−^ is a relatively stable intermediate which will become an unstable anion radical NO_2_^2−^ through a direct electron transfer reaction pathway (Equation (7)) [29]. Similarly to NO_3_^2−^, NO_2_^2−^ will be rapidly hydrolyzed into adsorbed NO_ad_ through Equation (8) (*k* = 1.0 × 10^5^ s^−1^) [30].

NO_2_^−^→NO:

(7) $${{NO}_{2}^{-}}_{ad}+e^{-}\to {{NO}_{2}^{2-}}_{ad}\;E^{0}=-0.47\;V\;vs.\;SHE$$

(8) $${{NO}_{2}^{2-}}_{ad}+H_{2}O\to {NO}_{ad}+2{OH}^{-}\;k=1\times 10^{5}s^{-1}$$

NO_ad_ is the key intermediate species that determines the selectivity of the final products, N_2_ or NH_3_. In the Vooys–Koper pathway, NO_ad_ reacts with NO_aq_ in solution to form a short-term dimer byproduct, dinitrogen dioxide (HN_2_O_2_) (Equation (9)) [31], which is then reduced to dinitrogen oxide (N_2_O) by secondary electron transfer (Equation (10)) [23,32]. N_2_O can be further reduced to the unstable N_2_O^−^ (Equation (11)) [33]. This species will be readily transformed into the final product N_2_ (Equation (12)) [34].

NO→N_2_—Vooys–Koper pathway:

(9) $${NO}_{ad}+{NO}_{aq}+e^{-}+H^{+}\to {HN_{2}O_{2}}_{ad}\;E^{0}=0.0\;V\;vs.\;SHE$$

(10) $${HN_{2}O_{2}}_{ad}+e^{-}+H^{+}\to {N_{2}O}_{ad}+2H_{2}O\;E^{0}=1.59\;V\;vs.\;SHE$$

(11) $${N_{2}O}_{ad}+e^{-}\to {{N_{2}O}_{ad}}^{-}\;E^{0}=1.77\;V\;vs.\;SHE$$

(12) $${{N_{2}O}_{ad}}^{-}+e^{-}+2H^{+}\to N_{2}+H_{2}O$$

Based on the NO_3_^−^RR study on the Pt (100) crystal plane, researchers proposed the Duca–Feliu–Koper pathway, through which NO_3_^−^ will be electrochemically reduced to N_2_. In this pathway, stable NH_2ad_ will be generated by the reduction of NO_ad_ (Equation (13)) and coexists with NO_ad_ [35,36]. NO_ad_ and NH_2ad_ will recombine according to the Langmuir–Hinshelwood reaction to generate the transient substance nitrosoamide (NONH_2_) (Equation (14)), which will be rapidly decomposed to N_2_ (Equation (15)).

NO→N_2_—Duca–Feliu–Koper pathway:

(13) $${NO}_{ad}+{3H}_{2}O+4e^{-}\to {NH_{2}}_{ad}+4OH^{-}$$

(14) $${NO}_{ad}+{NH_{2}}_{ad}\to NON{H_{2}}_{ad}$$

(15) $$NON{H_{2}}_{ad}\to N_{2}+H_{2}O$$

Among the mechanism studies for NH_3_ formation, the electrochemical–electrochemical (EE) reaction mechanism consisting of several sequential direct electron-transfer reactions is the main pathway [31]. NO successively undergoes reductive hydrogenation to several intermediates, forming nitroxyl (HNO) (Equation (16)) [37], H_2_NO (Equation (17)) [31], and hydroxylamine (H_2_NOH) (Equation (18)) [38]. Hydroxylamine will be protonated (Equation (19)) and then rapidly electrochemically reduced into NH_3_ (Equation (20)) [39].

NO→NH_3_:

(16) $${NO}_{ad}+H^{+}+e^{-}\to {HNO}_{ad}\;E^{0}=-0.78\;V\;vs.\;SHE$$

(17) $${HNO}_{ad}+H^{+}+e^{-}\to {H_{2}NO}_{ad}\;E^{0}=0.52\;V\;vs.\;SHE$$

(18) $${H_{2}NO}_{ad}+H^{+}+e^{-}\to {H_{2}NOH}_{ad}\;E^{0}=0.90\;V\;vs.\;SHE$$

(19) $$H_{2}NOH+H^{+}⇌{H_{3}NOH}^{+}\;pK_{a}=5.92$$

(20) $${H_{2}NOH}^{+}+{2H}^{+}+2e^{-}\to NH_{3}+H_{2}O\;E^{0}=0.42\;V\;vs.\;SHE$$

In addition to the above direct reduction pathway, another hydrogenation pathway using adsorbed active hydrogen species (H_ad_) as a reductant is considered to be an important pathway to promote the production of NH_3_ [40,41]. In the potential region where hydrogen adsorption occurs on the Pt catalyst, the formation of NH_3_ is highly promoted [42], since H_ad_ is stable on the electrode surface in that potential window. H_ad_ will efficiently deoxygenate NO_3_^−^ and then hydrogenate the intermediates into NH_3_. A hydrogenation mechanism is postulated according to the theoretical calculation results (Equations (21)–(26)): [43]

(21) $${{NO}_{3}^{-}}_{ad}+2H_{ad}\to {{NO}_{2}^{-}}_{ad}+H_{2}O$$

(22) $${{NO}_{2}^{-}}_{ad}+H_{ad}\to {NO}_{ad}+{OH}^{-}$$

(23) $${NO}_{ad}+{2H}_{ad}\to N_{ad}+H_{2}O$$

(24) $$N_{ad}+H_{ad}\to {NH}_{ad}$$

(25) $${NH}_{ad}+H_{ad}\to {NH_{2}}_{ad}$$

(26) $${NH_{2}}_{ad}+H_{ad}\to {NH_{3}}_{ad}$$

However, how H_ad_ participates in the NO_3_^−^RR process is still not clear, and the key intermediates of the reaction have not been experimentally proved. The intrinsic mechanism of the hydrogenation pathway remains to be further explored.

### 2.2. Indirectly Autocatalytic Reduction Pathway

The indirectly autocatalytic reduction pathway occurs only in the strongly acidic solution of high NO_3_^−^ concentrations (1–4 mol L^−1^) with the presence of HNO_2_ [23] and mainly affects the step involving the reduction of HNO_3_ to HNO_2_. In this pathway, NO_3_^−^ itself is not the electroactive species and does not participate in the electron transfer process. NO_3_^−^ and HNO_2_^−^ will be transformed into NO_2_ or NO^+^ that is postulated as the electroactive species. With the accumulation of electroactive intermediates, the reaction will be highly enhanced. These two active substances can be generated via the Vetter pathway and the Schmid pathway, respectively (Equations (27)–(34)) [19]:

Vetter pathway:

(27) $${NO_{2}}^{-}+H^{+}⇌HNO_{2}$$

(28) $$HNO_{2}+HNO_{3}⇌N_{2}O_{4}+H_{2}O$$

(29) $$N_{2}O_{4}⇌2NO_{2}$$

(30) $$NO_{2}+e^{-}\to {NO_{2}}^{-}$$

Schmid pathway:

(31) $$HNO_{2}+H^{+}⇌{NO}^{+}+H_{2}O$$

(32) $${NO}^{+}+e^{-}\to NO$$

(33) $$N_{2}O_{4}+2NO+2H_{2}O⇌4HNO_{2}$$

(34) $$HNO_{3}+2NO+H_{2}O⇌3HNO_{2}$$

The following pathway of HNO_2_ to NH_3_ is similar to the direct reduction pathway mentioned previously. The indirect autocatalytic reduction mechanism could effectively accelerate the reaction rate of NO_3_^−^RR, which plays an important role in accelerating the conversion rate and reducing the reaction cost.

## 3. Research Status of Electrocatalytic NO_3_^−^RR to NH_3_ Production

### 3.1. Evaluation Criteria for NO_3_^−^RR Performance

Since nitrogen oxides and NH_3_ widely exist in air and water, N species pollution during the tests must be strenuously avoided to guarantee the reproducibility of the research results. MacFarlane and Ib Chorkendorff et al. [44,45] proposed a set of testing procedures for NH_3_ detection during NRR to ensure the accuracy of experimental results. The interference of N species pollution must be eliminated, and it must be determined that N in the formed NH_3_ is derived from N_2_ rather than from pollution from other N sources (catalysts, electrolytes, air, etc.) [11]. Similarly, the source of N in the formed NH_3_ in the NO_3_^−^RR process should also be strictly verified to obtain the real experimental results. Therefore, we need to have a unified understanding of the performance evaluation criteria for NH_3_ synthesis during the NO_3_^−^RR process.

There are two main factors affecting the NO_3_^−^RR test results: (1) pollution from other N sources and (2) the accuracy of the NH_3_ detection method. The pollution from other N sources mainly comes from air, reaction devices, electrolytes, and catalysts. Strict requirements on the cleanliness of the experimental equipment and accessories are essential to absolutely exclude these contaminations. Gases and chemicals used in experiments need to be at least analytically pure. The N element in the catalyst is another common contaminant affecting the experimental results. Many researchers who study single-atom catalyst systems should pay special attention to this if they use the MNC as the single-atom catalyst (M is the metal). It is necessary to verify whether the N content in the catalyst is consistent before and after the reaction. In addition, the ^15^N isotope labeling experiment is also one of the methods of confirming that the ^15^N in the product ^15^NH_3_ comes from ^15^NO_3_^−^ instead of pollution from other N sources. The Macfarlane and Zhang groups [46,47] proposed strict experimental methods for the ^15^N isotope labeling experiment for NRR. Quantitative detection methods for NH_3_ include UV–Vis spectroscopic/colorimetric methods (Nessler reagent and indophenol blue method) [47,48], the o-phthalaldehyde fluorescence method [49], ion chromatography (IC) [50], ion selective electrode (ISE) [51], hydrogen nuclear magnetic resonance spectroscopy (^1^H NMR) [46,52], etc. The accuracy of these detection methods can be affected by environmental factors. For example, the pH will affect the complexation of the chromogenic agent with NH_3_, thereby affecting the coloration. The presence of other cations may affect the peak signal of NH_4_^+^ when using ion chromatography detection. Two different detection methods are usually required for a simultaneous detection to ensure the accuracy of the experimental results. This involves using isotope-labeled ^15^NO_3_^−^ as the N source for the reaction and then using ^1^H NMR to detect the ^15^NH_3_ product so that we can track the source of N, which is currently considered to be the safest measurement.

The production yield per unit area (mmol h^−1^ cm^−2^) and the production yield per unit mass (mmol h^−1^ g^−2^) are the key indicators in evaluating the NH_3_ production rate. Faradaic efficiency (%) and partial current density (mA cm^−2^) are important indicators for the measurement of the energy utilization efficiency of the electrocatalytic process. In addition, the overpotential (V vs. RHE), stability, and NO_3_^−^ removal rate (%) of the reaction are also important factors in evaluating the overall catalytic performances.

### 3.2. Electrocatalysts Designed for NO_3_^−^RR to NH_3_

**Figure 3:**
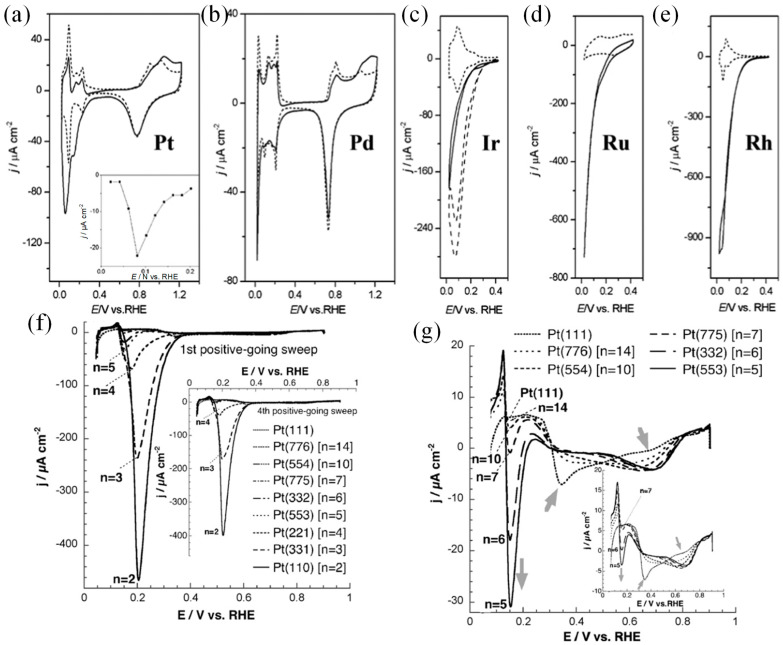
Cyclic voltammograms on (**a**) Pt, (**b**) Pd, (**c**) Ir, (**d**) Ru, and (**e**) Rh in 0.5 M H_2_SO_4_ with (solid line) or without (dashed line) the presence of 0.1 M NaNO_3_. Inset in (**a**) is the steady-state NO_3_^−^ reduction current at different potentials [20]. (**f**) Cyclic voltammograms at Pt(1 1 1) and Pt(S)[*n*(111) × 111]] electrodes (*n* = 14, 10, 7, 6, 5, 4, 3, 2) in 0.1 M 10 mM KNO_3_ + HClO_4_. (**g**) The cyclic voltammograms of the expansion of current density scale of (**f**) [42].

Noble metal catalysts have shown good electrocatalytic performances in many electrocatalytic reactions, such as hydrogen evolution, alcohol oxidation, nitrogen reduction, and carbon dioxide reduction reactions. In their study of electrocatalytic NO_3_^−^RR, DIMA et al. [20] analyzed the catalytic performance of the Pt group catalysts and found that the corresponding NO_3_^−^RR activity decreased following the sequence Rh > Ru > Ir > Pd > Pt (Figure 3a–e). The adsorption of NO_3_^−^ over Pt and Pd is weak, while the adsorption of *H is too strong, making it difficult for NO_3_^−^RR to compete with HER. Rh, Ru, and Ir have a higher ability to adsorb NO_3_^−^ and so those catalysts show better catalytic activity. Li et al. [53] reported that nitrate electroreduction overstrained Ru nanoclusters gave a high rate of NH_3_ formation (5.56 mol g_cat_^−1^ h^−1^). The primary contributor to such performance is the *H, which are generated by suppressing H-H dimerization during the water splitting enabled by the tensile lattice strains. The *H expedited NO_3_^−^-to-NH_3_ conversion by hydrogenating intermediates of the rate-limiting steps at lower kinetic barriers. The strained nanostructures can maintain nearly 100% NH_3_ selectivity at >120 mA cm^−2^ current densities for 100 h. Feliu et al. [42] found that electrocatalytic nitrate reduction was hardly perceptible on Pt (111), while electrocatalytic activity was improved with an increase in the step density of the Pt electrode surface. Inactivation was observed on the step sites in the presence of adsorbed NO_3_^−^-derived reduced adsorbates, i.e., adsorbed NO. It was, therefore, concluded that the electrocatalytically active NO_3_^−^ species did not adsorb on the (111) terraces but on the (111) monoatomic steps (as shown in Figure 3f,g). In addition, Han et al. [54] prepared Pd nanocrystalline with well-designed facets that could act as an efficient NO_3_^−^RR electrocatalyst for ambient NH_3_ synthesis and found that Pd (111) exhibited excellent activity and selectivity in reducing NO_3_^−^ to NH_3_ with a Faradaic efficiency of 79.91% and an NH_3_ production of 0.55 mmol h^−1^ cm^−2^ (2.74 mmol h^−1^ mg^−1^) in 0.1 M Na_2_SO_4_ (containing 0.1 M NO_3_^−^), which was 1.4 times higher than Pd (100) and 1.9 times higher than Pd (110), respectively. However, although many studies have been conducted on noble metals and some interesting results have been obtained, more effort should be made to lower the cost of the fabrication of catalysts and apply them to the industrial production of NH_3_ formation. Therefore, many researchers have turned their attention to the development and design of non-precious metal catalysts.

**Figure 4:**
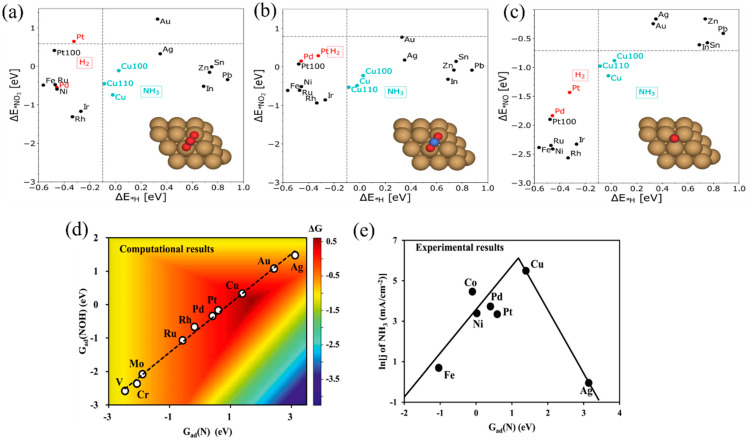
The adsorption energies of the intermediates (**a**) $∆E_{{*NO}_{3}}$; (**b**) $∆E_{{*NO}_{2}};$ and (**c**) $∆E_{*NO}$ corresponding to ΔE_*H_ [55]. (**d**) A two-dimensional activity map for producing NH_3_, where all the reaction free energies are shown at 0 V vs. RHE [12]. (**e**) The logarithm of partial current density of NH_3_ for NORR on different metal electrodes at 0 V vs. RHE [56].

Many transition metal catalysts exhibit a good electrocatalytic activity for NO_3_^−^RR. Rossmeissl et. al. [55] studied the thermodynamics of various transition metal catalysts based on DFT calculations and found that Cu has a good adsorption capability for the intermediate species such as *NO_3_^−^, *NO_2_^−^, and *NO (Figure 4a–d), which is conducive to the continuous reduction and transformation of NO_3_^−^. In addition, the adsorption of *H over Cu is weak, which hinders HER competition. Compared with other catalysts (as shown in Figure 4e), *N on Cu is more easily hydrogenated to NOH, which indicates that it has a better NH_3_ selectivity [12,56]. Jiao et al. [57] experimentally investigated various transition metals (Fe, Co, Ni, Cu) for the electrochemical reduction of NO and N_2_O to reveal the role of electrocatalysts in determining the product selectivity. Specifically, Cu was highly selective toward NH_3_ formation with >80% Faradaic efficiency in NO electroreduction. Furthermore, a high NO coverage facilitated the N–N coupling reaction. In acidic electrolytes, the formation of NH_3_ is greatly favored, whereas N_2_ production is suppressed.

## 4. Cu-Based Catalyst for NO_3_^−^RR

### 4.1. Modifications for Cu-Based Catalysts

Currently, the NH_3_ production rate over Cu-based catalysts is far lower than the traditional Haber–Bosch method (200 mmol g_cat_^−1^ h^−1^), which hinders its further development in industrial applications. Due to the complex reaction process of NO_3_^−^RR to NH_3_ and the low kinetics of NO_3_^−^RR, the current density of NO_3_^−^ reduction over Cu-based electrocatalysts is mostly below 100 mA cm^−2^, especially in solutions with a low NO_3_^−^ concentration. Therefore, it is urgent to improve the intrinsic activity of Cu-based catalysts for NH_3_ formation. Research published in the literature describes the following methods of modification for Cu-based catalysts, which have been studied extensively; they are summarized below.

#### 4.1.1. Size Modulation

**Figure 5:**
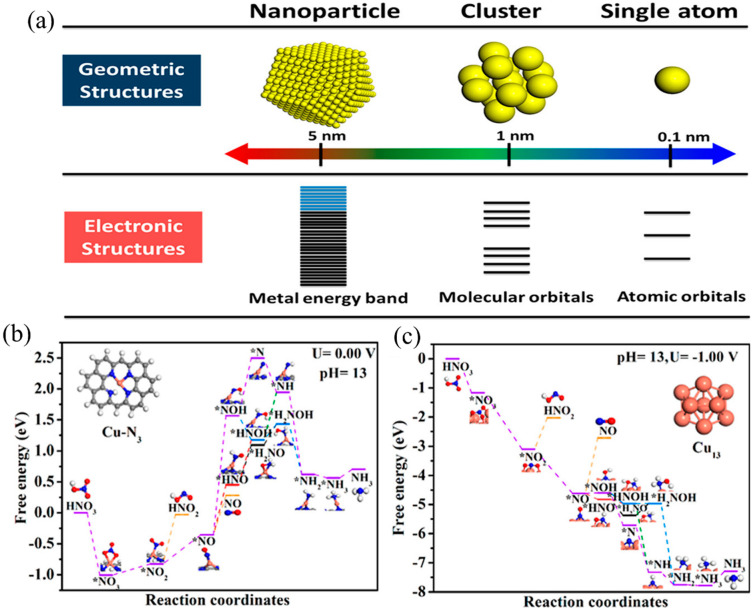
(**a**) Schematic diagrams of the geometric structures and electronic structures of single atoms, clusters, and nanoparticles [58]. Free energy diagrams of the electrochemical NO_3_^−^RR to NO_2_^−^/NH_3_ on (**b**) Cu-N_3_ and (**c**) Cu_13_ sites [61].

The catalytic activity of the catalyst is highly affected by its particle size [58]. By optimizing the particle size of the catalyst, the catalytic activity of the catalyst can be significantly improved. As shown in Figure 5, from nanoparticles to nanoclusters and even single-atom catalysts, the density of active sites per unit mass increases, normally giving an increased catalytic activity [58]. Fu et al. [59] reported that the current density of NO_3_^−^ reduction over Cu nanoparticles is 13 times higher than that over Cu foil, and the Faraday efficiency of NH_3_ production has also increased from 30% over Cu foil to approximately 61% over Cu nanoparticles. In addition, the particle size of the catalyst will affect the interaction of reactants with the catalyst’s surface [60]. Yang et al. [61] prepared a Cu single-atom catalyst (Cu SAC) for NO_3_^−^RR during which the maximum NH_3_ yield reached 0.26 mmol cm^−2^ h^−1^ (12.5 mol g_Cu_^−1^ h^−1^) and the Faraday efficiency of NH_3_ production reached 84.7%. According to in situ X-ray absorption spectroscopy coupled with advanced electron microscopy technology, it was found that Cu SAC would be reconstructed into Cu nanoparticles (Cu_13_) with a particle size of approximately 5 nm during NO_3_^−^RR. DFT calculation results also showed that there was a high thermodynamic energy barrier of *NO_2_ reduction over Cu SAC, while the thermodynamic energy barrier of *NO_3_ to NH_3_ on a Cu_13_ cluster presented a continuous downward trend. These results showed that it was the Cu nanoparticles that were the active sites and not the Cu single atom sites.

#### 4.1.2. Crystalline Facet Engineering

**Figure 6:**
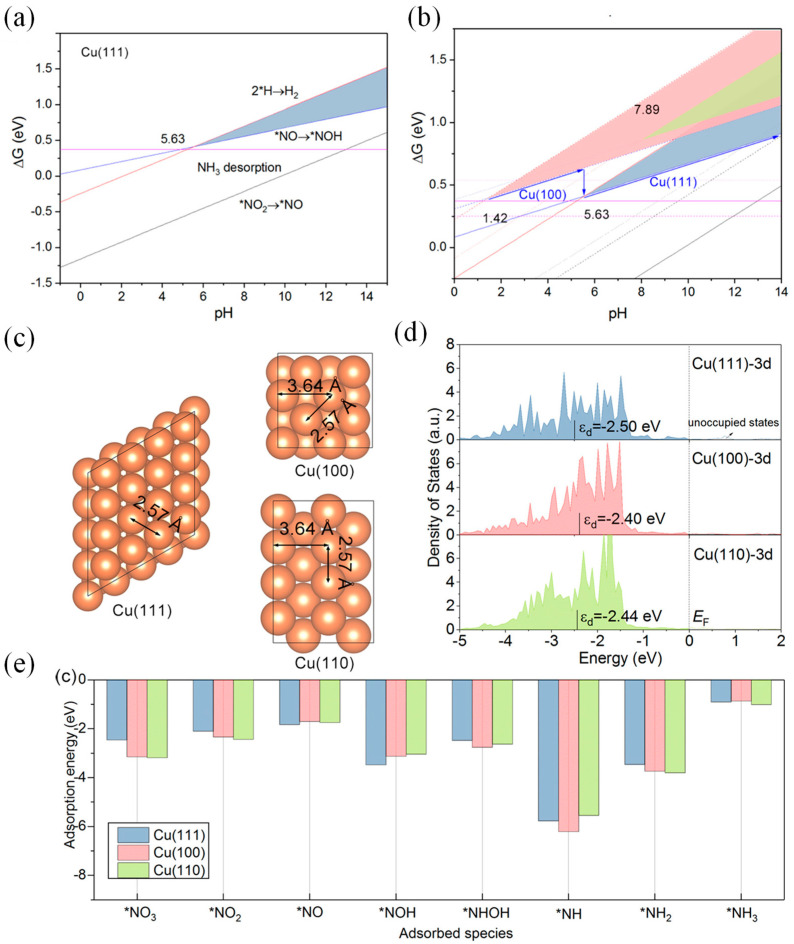
Competing relationship between NRA and HER on (**a**) Cu (111) and (**b**) comparison between Cu (111), Cu (110) and Cu (100). (**c**) Surface structure, (**d**) d-band centers of Cu (111), Cu (120), and Cu (100), and (**e**) intermediate adsorption energies on Cu (111), Cu (110), and Cu (100) [44].

**Figure 7:**
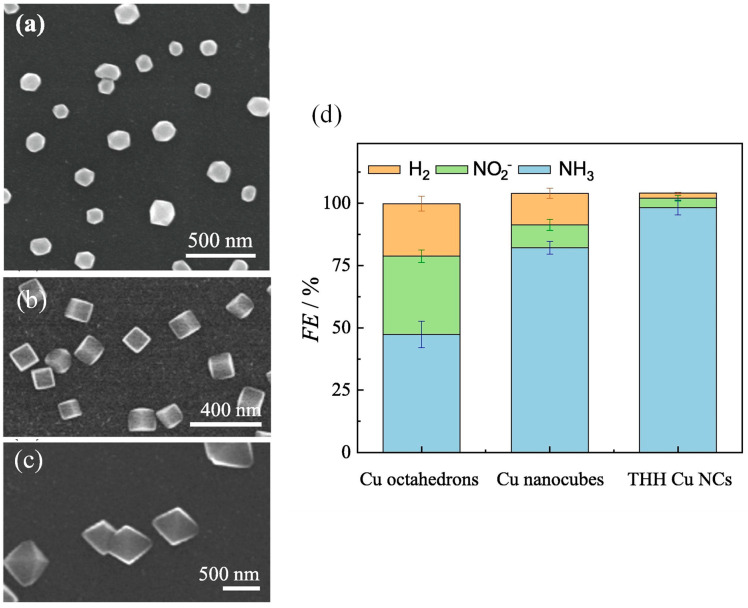
SEM images of (**a**) THH Cu NCs, (**b**) Cu nanocubes, and (**c**) Cu octahedrons; (**d**) FE of NH_3_ on THH Cu NCs, Cu nanocubes and Cu octahedrons [64].

Feliu and Koper [42,62] found that NO_3_^−^RR on the Pt-based catalyst was highly affected by the surface crystalline facet. Recent NO_3_^−^RR production also proved that NO_3_^−^RR on the Cu-based catalyst was sensitive to the crystalline facet. Hu et al. [44] studied the NO_3_^−^RR and HER processes on different basal facets of Cu by using theoretical calculations and found that the competition between NO_3_^−^RR and HER was affected by the solution pH. Cu (100) showed a catalytic performance of NO_3_^−^ reduction to NH_3_ in a strong acidic environment that was higher than that of the other basal facet, while Cu (111) had a higher catalytic activity in a neutral or alkaline environment (Figure 6). The difference in NO_3_^−^RR activities between the different crystalline facets of Cu are attributed to the different atomic spacing and electronic state of the surface atom. The Cu–Cu bond length on Cu (111) was closer to the distance of two oxygen atoms in NO_3_^−^, which is more conducive to the adsorption of NO_3_^−^ on Cu (111). In addition, in the theoretical calculation, the d-band center on the Cu (111) was higher than that of Cu (100), due to which there was a stronger adsorption ability to NO_3_^−^ on Cu (111). The electronic state of the catalyst surface affected its adsorption energy to NO_3_^−^ and other intermediate species that affected the NO_3_^−^RR activity. At a potential of 0 V vs. RHE, *NO→*NOH was theoretically proved as the rate-limiting step. The low adsorption energy of *NOH can reduce the reaction energy barrier. Fu et al. [59] prepared the Cu catalysts with different basal facets and found that Cu (111) exhibited its highest catalytic activity in alkaline electrolytes. Koper et al. [63] studied the performance of the electrocatalytic NO_3_^−^ over Cu single crystal electrode in different pH media. In acidic and alkaline media, the onset potential of Cu (111) was slightly more positive than Cu (100). However, the HER activity on Cu (111) was relatively higher than Cu (100), leading to better NO_3_^−^RR performances over the latter crystalline facet within a wide electrode potential window. Based on the advantages of electrochemically controllable technology in nanocrystals synthesis, our research group prepared Cu nanocrystals with different crystal plane, including high-index faceted Cu tetrahexahedron (THH Cu NCs, mainly composed of (211) and (311) crystal planes), low-index faceted Cu cube (100), and Cu octahedron (111) [64]. We found that, compared with Cu cube (100) and Cu octahedron (111), the THH Cu NCs with higher surface stepped sites had a higher reduction current density and a higher Faraday efficiency of NH_3_ formation of 98.3% (Figure 7). This is consistent with the results on the Pt catalyst reported by Feliu et al. [42]. The surface stepped sites of the catalyst are the main active center. The higher the density of the steps, the better the NO_3_^−^RR activity.

#### 4.1.3. Surface Defects Engineering

By constructing the defective structure and decreasing the coordination number of the active site on the catalysts surface, NO_3_^−^ adsorption and activation on the surface can be significantly enhanced and the intrinsic activity of the catalysts can then be improved. Hu et al. [65] studied the effect of defect exposure on the surface of Cu (100) on NO_3_^−^ reduction activity. They found that under alkaline conditions, ultra-thin two-dimensional CuO (111) nanoribbons reconstituted into Cu (100) nanoribbons (Cu-NBS -100) in the process of NO_3_^−^ electrochemical reduction, and, due to the high surface energy of ultra-thin nanoribbons, certain surface defects were formed in the process of structure reconstruction. Cu (100) -D1~7 models with different defects were constructed for DFT calculation. After comprehensive study, it was found that the d-band center of Cu (100)-D upshifted towards the Fermi level after the construction of surface defects on which the adsorption of intermediate species was enhanced. The Gibbs free energy of Cu (100)-D7 for NO_3_^−^ was significantly lower than that of Cu (100). The defective sites on Cu (100)-D7 had a strong adsorption effect on *H intermediates, which makes *H difficult to desorb from the Cu surface, thus inhibiting HER. Xu et al. [66] also proved that Cu nanosheets with rich surface defects had a higher catalytic activity than those with smooth surfaces. At the potential of −1.3 V vs. SCE, the production rate of NH_3_ reached 781.25 μg h^−1^ mg^−1^, and the Faraday efficiency arrived at 81.99%. Surface defects were able to change charge distribution on the catalyst surface and effectively improved electron transfer between the catalyst and the adsorbed species. They were able to enhance the adsorption and conversion of reaction intermediates on the catalyst surface [67]. In addition, surface atomic defects also brought more irregular edge steps to the catalyst surface and increased the density of surface-active sites.

#### 4.1.4. Interfacial Engineering

In photocatalysis, constructing heterojunction structures to apply interfacial interactions to improve photocatalytic activity is a common catalyst-design strategy [68,69,70]. In electrocatalysis, the interfacial interaction between different phases also has a significant impact on catalytic performances [71,72]. Deng et al. [73] proved that the electrocatalytic NO_3_^−^RR activity of CoP can be effectively enhanced by constructing a p-n heterojunction of a CoP/TiO_2_ nanoarray on titanium plate (CoP/TiO_2_@TP). According to DFT, the p-n heterojunction in CoP/TiO_2_ could induce the charge redistribution of CoP and TiO_2_, enhance the electron transport between the interfaces, effectively reduce the free energy of each reaction step, and improve the performance of NO_3_^−^RR. Benefiting from the interaction of heterostructures, such CoP/TiO_2_@TP exhibited an excellent Faradaic efficiency of 95.0% and a large NH_3_ yield as high as 499.8 μmol h^−1^ cm^−2^, which was superior to CoP@TP and TiO_2_@TP. For a Cu-based catalyst, Liu et al. [74] grew Pd particles on CuO nano-olives (Pd/CuO NOs) to form a Pd/CuO NOs catalyst with a unique Pd/CuO heterogeneous interface for nitrite reduction (NO_2_^−^RR). The heterogeneous interface formation of Pd/CuO NOs was analyzed by means of XPS characterization and DFT calculation. CuO and Pd show the characteristics of negative charge and positive charge, respectively, because of the transfer of electrons. Additionally, the built-in electric field constructed at the Pd/CuO heterointerface promotes local charge polarization. The formation of a Pd/CuO heterointerface optimizes the electronic structure of Cu, which is more favorable for the adsorption of NO_2_^−^ and reaction intermediates. Pd/CuO NOs showed an NH_3_ yield of 906.4 μg h^−1^ mg^−1^_cat_ and a Faraday efficiency of 91.8%. Wang et al. [75] designed the Cu/Cu_2_O nanowire array and revealed that CuO would be electrochemically converted into Cu/Cu_2_O, which was observed by performing in situ Raman and ex situ experiments. The Cu/Cu_2_O interface was proved as the active site. The combined results of online differential electrochemical mass spectrometry (DEMS) and DFT calculations demonstrated that the electron transfer from Cu_2_O to Cu at the interface could facilitate the formation of *NOH intermediate and suppress the HER, leading to 95.8% of NH_3_ Faraday efficiency and 0.2449 mmol h^−1^ cm^−2^ NH_3_.

In summary, the optimization of Cu catalysts by various modification methods can significantly improve the Faraday efficiency of NH_3_ production, but it was also found that the current density of NO_3_^−^RR and the NH_3_ production rate were still too low to be applied in industrial applications (Table 1). Moreover, the required overpotential was also too high and was not conducive to being powered by regeneration energy. To this end, researchers have gradually designed Cu-based bimetallic catalysts by introducing second metals, and tried to further improve the catalytic activity of Cu-based bimetallic catalysts by regulating their electronic structure and synergistic effect, and they have promoted the further development of NO_3_^−^RR.

#### 4.1.5. Electronic Structure Modulation

Wang et al. [82] designed Cu_100−x_Ni_x_ alloy with different proportions of the Cu and Ni elements and investigated the adsorption energy of intermediate products over the Cu_100−x_Ni_x_ alloy. Since the NO_3_^−^RR activity on Ni is weak, the increase in the Ni element proportion should decrease the number of active sites on the surface, possibly resulting in a decreased performance. However, compared with pure Cu, Cu_50_Ni_50_ showed an increase of 0.12 V in the half-wave potential and a sixfold increase in activity at 0 V vs. RHE. The electronic structure of Cu_100−x_Ni_x_ was analyzed by performing X-ray photoelectron spectroscopy (XPS), X-ray adsorption spectroscopy (XAS), and ultraviolet photoelectron spectroscopy (UPS). It was found that the binding energy of Cu 2*p* in Cu_100−x_Ni_x_ alloy shifted to a lower binding energy with the increase in the Ni proportion. The binding energy of the Ni 2*p* shifted towards a higher binding energy, indicating that the charge in the Cu_100−x_Ni_x_ alloy was redistributed and electrons were transferred from the Ni to the Cu. At the same time, the Cu d-band center upshifted, which enhanced the adsorption of the intermediate products on the Cu. Further study by means of DFT calculation found that the adsorption energy of the intermediate products showed a volcanic distribution with the overall reactivity. When the Ni accounted for 50%, the catalyst had moderate adsorption on all intermediate species, and then the highest reaction performances arrived. The establishment of the relationship between the electronic structure of the catalysts and NO_3_^−^RR activity provided an idea for guiding the design of novel NO_3_^−^RR catalysts. Ge et al. [83] reported polyallylamine (PA) functionalized RhCu bimetallic nanocubes (PA-RhCu cNCs) as a robust electrocatalyst for the reduction of NO_3_^−^ to NH_3_. PA-RhCu cNCs showed a remarkable NH_3_ production yield of 2.40 mg h^−1^ mg_cat_^−1^ and a high Faradaic efficiency of 93.7% at 0.05 V. DFT calculations and experimental results indicated that Cu and PA (adsorbed amino) co-regulated the Rh d-band center, which slightly weakens the adsorption energy of reaction-related species on Rh. These findings may open an avenue to construct other advanced catalysts based on organic molecule-mediated interfacial engineering. Goldsmith’s group [84] predicted NO_3_^−^RR activity by calculating the adsorption energy of N and O atoms on transition metals. They proposed that alloyed Fe_3_Ru, Fe_3_Ni, Fe_3_Cu, and Pt_3_Ru have a strong adsorption capability to N and O atoms and suggested that those bimetals should be the potential NO_3_^−^ reduction catalysts.

Based on the above analysis, the appropriate adsorption energy of catalysts for intermediate species such as *NO_3_, *H, and *NH_3_ is the key factor affecting their performance, which can be achieved by modulating the electronic structure of bimetal alloys to affect the d-band center or by forming a poor/rich-electron center.

#### 4.1.6. Synergistic Effect

Protons (8e^−^) are involved in the process of NO_3_^−^ reduction to NH_3_, giving a series of elementary reactions before the final products are formed. The active sites on single metallic catalysts are not well adapted to simultaneous multiple elementary steps. To this end, the construction of a synergistic effect between multiple active sites become one of the research hotspots in the field of NO_3_^−^RR. Precious metals alloying with non-precious metals is one of the most useful strategies for fabricating bimetallic catalysts (Table 2). The introduction of precious metals into Cu metal was proved to facilitate a much higher catalytic activity than that of pure Cu [85,86,87,88]. Kerkeni et al. [89] modified the platinum catalyst’s surface through a deposition on the Cu (sub)monolayer of a certain amount of Cu (Cu_ads_/PT) using electrochemical methods. They found that the intrinsic catalytic activity (at *t* = 0) and the NH_3_ selectivity of Cu_ads_/Pt depended on the fraction of the platinum surface occupied by copper adatoms (*θ*_Cu_). The highest activities and selectivity of NH_3_ were obtained with *θ*_Cu_ ≈ 0.4–0.6. Moreover, higher activities were obtained in the reduction of NO_3_^−^ than in the reduction of NO_2_^−^. Barrabés [85] and Wang et al. [90] believed that the existence of Cu was conducive to NO_3_^−^ adsorption and reduction into NO_2_^−^ and that NO_2_^−^ would then be transferred to Pt/Pd to continue being hydrogenated. The presence of Pt/Pd would produce *H, which was conducive to maintaining the existence of Cu^0^ activity and promoting the intermediate species to further hydrogenation to N_2_ or NH_3_. Luo et al. [91] designed the Cu nanowire-supported Rh cluster and single-atom electrocatalyst (Rh@Cu) for a NO_3_^−^ reduction reaction. Benefiting from the catalytic cooperation between Rh and Cu, whereby Rh activates the hydrogenation of the *NO intermediate on Cu, Rh@Cu systems achieved a Faradaic efficiency up to 93% at −0.2 V vs. an RHE and NH_3_ yield rate of 1.27 mmol h^−1^ cm^−2^. Detailed investigations by means of electron paramagnetic resonance, in situ infrared spectroscopy, differential electrochemical mass spectrometry, and DFT calculations suggested that the high activity originates from the synergistic cooperation between the Rh and Cu sites, whereby adsorbed hydrogen on the Rh site transferred to vicinal *NO intermediate species adsorbed on the Cu. Schuhmann et al. [92] presented a design concept of tandem catalysts, which involves coupling intermediate phases of different transition metals, existing at low applied overpotentials, as cooperative active sites that enable cascade NO_3_^−^-to-NH_3_ conversion, in turn, avoiding the generally encountered scaling relations. They implemented the concept by means of the electrochemical transformation of Cu−Co binary sulfides into potential-dependent core−shell Cu/CuO_x_ and Co/CoO phases. Electrochemical evaluation, kinetic studies, and in situ Raman spectra reveal that the inner Cu/CuO_x_ phases preferentially catalyzed NO_3_^−^ reduction to NO_2_^−^, which was rapidly reduced to NH_3_ at the nearby Co/CoO shell. This unique tandem catalyst system led to a NO_3_^−^-to-NH_3_ Faradaic efficiency of 93.3 ± 2.1% in a wide range of NO_3_^−^ concentrations at pH 13, a high NH_3_ yield rate of 1.17 mmol cm^−2^ h^−1^ in 0.1 M NO_3_^−^ at −0.175 V vs. RHE, and a half-cell energy efficiency of ~36%, surpassing most previous reports.

### 4.2. Study on Mechanism of Electrocatalysis of NO_3_^−^RR Synthesis of NH_3_ by Cu-Based Catalyst

**Figure 8:**
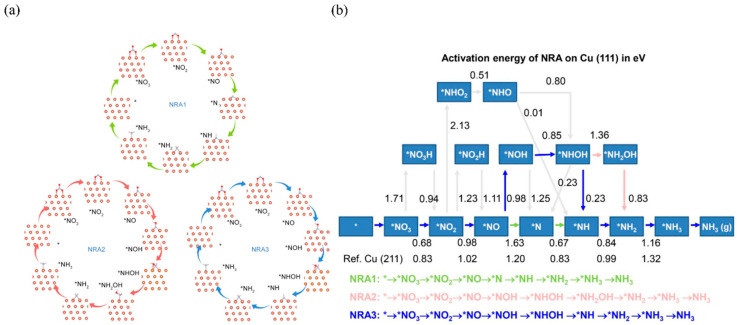
(**a**) The three possible NO_3_^−^RR pathways on Cu (111) and (**b**) the activation energy diagram of each step of NO_3_^−^RR synthesis of NH_3_ on Cu (111) [44].

Hu et al. [44] studied the pathway of the electroreduction of NO_3_^−^ to NH_3_ by employing DFT calculation. According to their comprehensive thermodynamic and dynamic analysis, the pathway of NO_3_^−^RR on Cu should be as follows: NO_3_^−^ → *NO_3_ → *NO_2_ → *NOH → *NHOH → *NH → *NH_2_ → *NH_3_ → NH_3_ (g) (Figure 8). Butcher et al. [99] detected a series of intermediates in the NO_3_^−^RR process in the acid media by means of SHINERS analysis, including NO_2_^−^ and HNO, NH_2_, NH_4_^+^, etc. This was consistent with DFT calculation results. In an alkaline medium, Koper et al. [63] observed the appearance of hydroxylamine on the Cu catalyst using Fourier infrared spectra analysis, indicating that hydroxylamine was an intermediate species in the alkaline environment.

Gewirth et al. [100] analyzed the structural evolution of the adsorption intermediates on the surface of the Cu (100) by using an in situ electrochemical scanning tunnel microscope (EC-STM). They intuitively observed the process of adsorption of NO_3_^−^ being transformed into NO_2_^−^. The adsorption of oxyanions was always accompanied by the synergistic adsorption of H_2_O or H_3_O^+^ [101]. H_2_O or H_3_O^+^ can stabilize the structure of oxyanions by forming hydrogen bonds with the lone electron pairs of oxygen atoms [102]. Researchers proposed that the NO_3_^−^ adsorption layer on Cu (100) was also involved with H_3_O^+^. In addition, DFT calculation results showed that NO_3_^−^ and NO_2_^−^ were adsorbed on the Cu (100) surface by the bridging of their two oxygen atoms. Koper et al. [20] conducted an electrochemically dynamic analysis of the NO_3_^−^RR process using cyclic voltammetry and found that the Tafel slope on the Cu-based catalyst was slightly higher than the 120 mV dec^−1^. Therefore, they proposed that the first electron transfer step was the rate-limiting step. There are two possible mechanisms: *H reduction (Equations (35) and (36)) and the proton-coupling electron reduction mechanism (Equations (37) and (38)).

*H reduction:

(35) $$H^{-}+e^{-}\to H_{ad}$$

(36) $${{NO}_{3}^{-}}_{ad}+H_{ad}\to adsorbed\;intermedia$$

Proton-coupling electron reduction:

(37) $${{NO}_{3}^{-}}_{ad}+H^{+}+e^{-}\to {{HNO}_{3}^{-}}_{ad}$$

(38) $${{HNO}_{3}^{-}}_{ad}\to adsorbed\;intermedia$$

The HER performance over the Cu catalyst is relatively poor, so it is difficult to produce *H species. Therefore, it is generally believed that the proton-coupling electron reduction mechanism is most effective on a pure Cu catalyst. For catalysts that are more susceptible to activating protons, such as the precious metals contained in bimetals, *H was more conducive to NO_3_^−^ reduction.

The existence of other anions/cations in electrolytes usually affects the target catalytic reactions. For example, the existence of Cl^−^ ions would lead to different final products. Butcher et al. [99], analyzing to Raman spectra, found that there were more bands of N-H_x_ species on the Cu basal facets after adding 10 mmol L^−1^ Cl^−^, revealing that the presence of Cl^−^ was beneficial for NH_3_ formation. Gao et al. [103] found that the amount of NH_3_ formation was dramatically decreased, although a similar removal efficiency of NO_3_^−^ was obtained when 100 mg L^−1^ Cl^−^ was added. As the Cl^−^ ion concentration increased, the NH_3_ selectivity rapidly decreased. This was due to the Cl^−^ indirectly oxidizing NH_3_ into N_2_. The Cl^−^ was oxidized by the anode to produce Cl_2_ (Equation (39)), which was then transformed into HOCl with strong oxidation (Equation (40)) [104]. The HOCl could efficiently oxidize the NH_3_ into N_2_ (Equation (41)).

(39) $$2{Cl}^{-}\to {Cl}_{2}+2e^{-}$$

(40) $${Cl}_{2}+H_{2}O\to HOCl+H^{+}+{Cl}^{-}$$

(41) $$3HOCl+2NH_{3}\to N_{2}+{3H}_{2}O+3{Cl}^{-}+3H^{+}$$

Currently, mechanistic studies of NO_3_^−^RR over Cu-based catalysts are relatively scarce, and most of them have followed the results from Pt-based catalysts. Therefore, the mechanism of electrocatalytic NO_3_^−^ reduction to NH_3_ over Cu-based catalysts requires further study.

## 5. Conclusions and Perspectives

Electrocatalytic NO_3_^−^RR is a clean and pollution-free process for NH_3_ production, which has the potential to realize large-scale industrial NH_3_ production and could solve the problem of NO_3_^−^ pollution at the same time. This review mainly presents contemporary views on the state of the art in electrocatalytic NO_3_^−^ reduction over Cu-based nanostructured materials. The effective methods, which were reported to modify the performance of Cu-based catalysts for electrocatalytic NO_3_^−^RR to NH_3_, include size modulation, crystalline facet engineering, surface defect engineering, interfacial engineering, electronic structure modulation, and synergistic effect. A reduction in metal catalyst particle size can effectively improve the utilization efficiency of atoms and enhance reactivity. Theoretically, the single-atom catalyst has nearly 100% atomic utilization efficiency. However, for Cu single-atom catalysts, the active sites were Cu nanoparticles instead of the Cu single atom sites. For the optimization of crystal surfaces, Cu nanocrystalline catalysts with a high crystal index have higher activity than those with a low crystal index. Cu catalysts with a high crystal index can be constructed to further improve the performance of NH_3_ synthesis. Moreover, by constructing the defective structure and decreasing the coordination number of the active site on the catalyst’s surface, NO_3_^−^ adsorption and activation on the surface can be significantly enhanced, and the intrinsic activity of the catalysts can then be improved. Cu active sites can be optimized effectively by the three methods mentioned above. However, since the reduction of NO_3_^−^ to NH_3_ involves the transfer of nine protons and eight electrons, it is difficult for the single active site to work effectively with simultaneous multiple elementary steps. To this end, catalysts with multiple active sites were designed. The heterogeneous interface between the Cu-based catalysts and other materials could promote local charge polarization by means of the formation of a built-in electric field and change the adsorption of intermediate species of NO_3_^−^RR. The alloying of Cu with a secondary metal can regulate the electronic structure of Cu and the synergistic effect. Additionally, different elements/active sites with different activities may have synergistic effects by providing a reduction in intermediate species or tandem reaction mechanisms to effectively reduce the reaction energy barrier. In conclusion, there are broad development spaces in the design and development of catalysts for the electrocatalytic NO_3_^−^RR of synthetic NH_3_, and this paper can provide a reference for subsequent research.

The research of nitrate reduction reaction has developed vigorously and made some progress in recent years, but there are still many problems. Below, we list some of the shortcomings and further research directions.

Research on the mechanism of NO_3_^−^RR is still insufficient. The reaction pathway of NO_3_^−^RR is affected by many environmental parameters, such as other ions, pH, NO_3_^−^ concentration, and applied potential. Different systems accrue different basic pathways, which require a more systematic and detailed analysis, including the identification of key reaction intermediates by means of various in situ spectra and the postulation of basic reaction steps using theoretical calculations.

Electrocatalysts that are more economical, efficient, and stable need to be developed. At present, although modified Cu-based catalysts show improved electrocatalytic reduction activities, there are oxidation, dissolution, and leaching problems in catalysts, and these lead to the decay of electrochemical activity and could cause adverse effects on the environment. In addition, structural evolution during electrocatalysis makes it more difficult to identify the real active sites and design strategies for protecting these active sites. Various electrochemical in situ spectral analyses and electron microscopic observations can help track the evolution of active sites during electrocatalysis and provide guidance and assistance for catalyst design. Furthermore, the predictive power of machine learning based on the analysis of large experimental data will help to develop the rapid screening of suitable and efficient NO_3_^−^RR catalysts.

There are differences between the actual sewage and the experimental test electrolyte. The potential and limitations of electrocatalytic NO_3_^−^RR in practical water-remediation systems should be investigated by fully considering the effects of possible ion pollution, organic compound pollution, and solid sediment on NO_3_^−^RR in actual sewage and then designing the corresponding solutions. For example, pretreatment devices could be designed to purify the pollution.

It is necessary to design and build a reasonable and efficient reaction system and device. First, in order to form a complete chemical reaction process with NO_3_^−^RR, another set of electrocatalytic reactions (such as oxygen evolution) would be required, which would not only increase the complexity and cost of the reaction but also mean that the oxidation process and reaction byproducts might interfere with NO_3_^−^ reduction. Therefore, we need to optimize the electrolytic cell. In addition, it is necessary to overcome the influence of mass transfer on scale-up experiments. The design of more efficient flow electrolytic cells is also one of the keys to improving the efficiency of NO_3_^−^RR.

The design and evaluation of the whole chain of the NH_3_ synthesis process by electrochemical NO_3_^−^RR require further work. At present, the low efficiency of NH_3_ extraction is an important factor hindering the development of NH_3_ production by NO_3_^−^RR. Therefore, the overall efficiency and the feasibility of NO_3_^−^RR in practical applications still need to be further evaluated. For example, the efficiency and cost of electrocatalytic NO_3_^−^RR technology and the Haber–Bosch process, as well as other important parameters related to scalability and commercial feasibility, should be investigated to make a comprehensive technological and economic evaluation. 

## Figures and Tables

**Table 1:** Recently reported Cu-based metallic catalysts for NO_3_^−^ reduction to NH_3_.

Cathode Material	FE to NH_3_	Current Density (mA cm^−2^)	NH_3_ Production Reported	Conditions	Potential (V vs. RHE)	Ref.
Cu foil	30%	~−0.36	3.9 ug_NH3_ mg_cat_^−1^ h^−1^ (3.9 ug_NH3_ h^−1^ cm^−2^)	10 mM KNO_3_, 0.1 M KOH	−0.15	[59]
Cu nanoparticle	61%	~−4.65	225.8 ug_NH3_ mg_cat_^−1^ h^−1^ (225.8 ug_NH3_ h^−1^ cm^−2^)	10 mM KNO_3_, 0.1 M KOH	−0.15	[59]
Cu nanosheets	99.7%	~−4.92	390.1 ug_NH3_ mg_cat_^−1^ h^−1^ (390.1 ug_NH3_ h^−1^ cm^−2^)	10 mM KNO_3_, 0.1 M KOH	−0.15	[59]
Cu SAC	84.7%	~−75	0.26 mmol cm^−2^ h^−1^ (12.5 mol g_Cu_^−1^ h^−1^)	0.1 M KNO_3_, 0.1 M KOH	−1.00	[61]
THH Cu NCs	98.3%	~−70	-	0.1 M K_2_SO_4_, 50.1 M KNO_3_	−0.9	[64]
Cu-NBs-100	95.3%	~−300	650 mmol g_cat_^−1^ h^−1^	0.1 M KNO_3_, 1 M KOH	−0.15	[65]
dr-Cu-NPs	81.99%	~−30	781.25 μg h^−1^ mg^−1^	0.5 M K_2_SO_4_, 50 ppm KNO_3_	−0.646	[66]
Cu-incorporated PTCDA	77%	-	0.0256 mmol h^−1^ cm^−2^	0.1 mM PBS, 36 mM NO_3_^−^	−0.4	[76]
Cu/Cu_2_O NWAs	95.8%	-	0.2449 mmol h^−1^ cm^−2^	0.5 M Na_2_SO_4_, 14.3 mM NO_3_^−^	−0.196	[75]
Cu crystallite	97%	~−6.4	-	0.1 M NaNO_3_, 1 M NaOH	−0.376	[77]
FOSP-Cu	93.9%	-	101.4 μmol h^−1^ cm^−2^	0.1 M KNO_3_, 0.5 M Na_2_SO_4_	−0.266	[78]
OD-Cu	92%	-	1.1 mmol cm^−2^ h^−1^	0.1 M KNO_3_, 1 M KOH	−0.15	[79]
BCN-Cu	98.2%	~−40	3358.74 μg h^−1^ cm^−2^	0.1 M KNO_3_, 1 M KOH	−0.6	[80]
Cu_2_O(100)	82.5%	-	743 μg h^−1^ mg_cat_^−1^	0.1 M NaSO_4_, 50 ppm NaNO_3_	−0.6	[81]

**Table 2:** Recently reported Cu-based bimetallic catalysts for NO_3_^−^ reduction to NH_3_.

Cathode Material	FE to NH_3_	Current Density (mA cm^−2^)	NH_3_ Production Reported	Conditions	Potential	Ref.
Cu_50_Ni_50_	99%	~−90	-	100 mM NO_3_^−^, 1 M KOH	−0.1V vs. RHE	[82]
Cu_70_Ni_3_0 porous	95.9%	~−400	-	0.1 M NaNO_3_, 1 M NaOH	−1.20 V vs. Hg/HgO	[93]
Cu_70_Zn_30_ porous	97%	~−400	-	0.1 M NaNO_3_, 1 M NaOH	−1.40 V vs. Hg/HgO	[94]
Au_1_Cu (111)	98.7%		555 μg h^−1^ cm^−2^	0.1 M KOH, 7.14 mM NO_3_^−^	−0.2 V vs. RHE	[95]
Cu_3_Pd_1_	90.02%		784.37 mg h^−1^ mg_cat_^−1^	0.5 M K_2_SO_4_, 50 ppm KNO_3_-N	−0.46 V vs. RHE	[96]
PdCu-Cu_2_O	94.32%		0.190 mmol h^−1^ cm^−2^	0.5 M Na_2_SO_4_, 100 ppm NO_3_^−^-N	−0.80 V vs. RHE	[97]
PA-RhCu cNCs	93.7%	-	2.40 mg h^−1^ mg_cat_^−1^	0.1 M HClO_4_, 0.05 M KNO_3_	+0.05 V vs. RHE	[83]
Rh@Cu	93.0%	−162	1.27 mmol h^−1^ cm^−2^	100 mM NO_3_^−^, 0.1 M Na_2_SO_4_	−0.2 V vs. RHE	[91]
CuCoSP	93.3%	−300	1.17 mol cm^−2^ h^−1^	100 mM NO_3_^−^, 0.1 M KOH	−0.175 V vs. RHE	[92]
CuCo nanosheet	100%	−1035	960 mmol h^−1^ mg_cat_^−1^	100 mM NO_3_^−^, 0.1 M KOH	0.4 V vs. RHE	[98]

## Data Availability

No new data were created or analyzed in this study. Data sharing is not applicable to this article.

## Abbreviations

Full name	Abbreviation
ammonia	NH_3_
nitrate	NO_3_^−^
nitrate reduction reaction	NO_3_^−^RR
nitrogen	N_2_
nitrogen reduction reaction	NRR
nitrite	NO_2_^−^
nitrogen monoxide	NO
nitrous oxide	N_2_O
dinitrogen dioxide	HN_2_O_2_
nitroxyl	HNO
hydroxylamine	H_2_NOH
nitrosoamide	NONH_2_
Renewable Energy to Fuels through Utilization of Energy-dense Liquids	REFUEL
Department of Energy	DOE
biocatalyzed by nitrate/nitrite reductase	bio-NRA
hydrogen evolution reaction	HER
adsorbed active hydrogen species	H_ad_
ion chromatography	IC
ion selective electrode	ISE
hydrogen nuclear magnetic resonance spectroscopy	^1^H NMR
single-atom catalyst	SAC
online differential electrochemical mass spectrometry	DEMS
X-ray photoelectron spectroscopy	XPS
X-ray adsorption spectroscopy	XAS
ultraviolet photoelectron spectroscopy	UPS
polyallylamine	PA

## References

[1] Fu X., Pedersen J.B., Zhou Y., Saccoccio M., Li S., Sažinas R., Li K., Andersen S.Z., Xu A., Deissler N.H. (2023). Continuous-flow electrosynthesis of ammonia by nitrogen reduction and hydrogen oxidation. Science.

[2] Erisman J.W., Sutton M.A., Galloway J., Klimont Z., Winiwarter W. (2008). How a century of ammonia synthesis changed the world. Nat. Geosci..

[3] Salmon N., Bañares-Alcántara R. (2021). Green ammonia as a spatial energy vector: A review. Sustain. Energy Fuels.

[4] MacFarlane D.R., Cherepanov P.V., Choi J., Suryanto B.H.R., Hodgetts R.Y., Bakker J.M., Ferrero Vallana F.M., Simonov A.N. (2020). A Roadmap to the Ammonia Economy. Joule.

[5] Kandemir T., Schuster M.E., Senyshyn A., Behrens M., Schlögl R. (2013). The Haber-Bosch process revisited: On the real structure and stability of “ammonia iron” under working conditions. Angew. Chem. Int. Ed..

[6] Nishina K. (2022). New ammonia demand: Ammonia fuel as a decarbonization tool and a new source of reactive nitrogen. Environ. Res. Lett..

[7] Van der Ham C.J., Koper M.T., Hetterscheid D.G. (2014). Challenges in reduction of dinitrogen by proton and electron transfer. Chem. Soc. Rev..

[8] Foster S.L., Bakovic S.I.P., Duda R.D., Maheshwari S., Milton R.D., Minteer S.D., Janik M.J., Renner J.N., Greenlee L.F. (2018). Catalysts for nitrogen reduction to ammonia. Nat. Catal..

[9] Chen G.-F., Cao X., Wu S., Zeng X., Ding L.-X., Zhu M., Wang H. (2017). Ammonia Electrosynthesis with High Selectivity under Ambient Conditions via a Li+ Incorporation Strategy. J. Am. Chem. Soc..

[10] DOE (2020). Renewable Energy to Fuels through Utilization of Energy-Dense Liquides.

[11] Suryanto B.H.R., Du H.-L., Wang D., Chen J., Simonov A.N., MacFarlane D.R. (2019). Challenges and prospects in the catalysis of electroreduction of nitrogen to ammonia. Nat. Catal..

[12] Long J., Chen S., Zhang Y., Guo C., Fu X., Deng D., Xiao J. (2020). Direct Electrochemical Ammonia Synthesis from Nitric Oxide. Angew. Chem. Int. Ed..

[13] Zeng Y., Priest C., Wang G., Wu G. (2020). Restoring the Nitrogen Cycle by Electrochemical Reduction of Nitrate: Progress and Prospects. Small Methods.

[14] Menció A., Mas-Pla J., Otero N., Regàs O., Boy-Roura M., Puig R., Bach J., Domènech C., Zamorano M., Brusi D. (2016). Nitrate pollution of groundwater; all right…, but nothing else?. Sci. Total Environ..

[15] Yao F., Jia M., Yang Q., Chen F., Zhong Y., Chen S., He L., Pi Z., Hou K., Wang D. (2021). Highly selective electrochemical nitrate reduction using copper phosphide self-supported copper foam electrode: Performance, mechanism, and application. Water Res..

[16] Ye S., Chen Z., Zhang G., Chen W., Peng C., Yang X., Zheng L., Li Y., Ren X., Cao H. (2022). Elucidating the activity, mechanism and application of selective electrosynthesis of ammonia from nitrate on cobalt phosphide. Energy Environ. Sci..

[17] Sundararajan M., Hillier I.H., Burton N.A. (2007). Mechanism of Nitrite Reduction at T_2_Cu Centers:  Electronic Structure Calculations of Catalysis by Copper Nitrite Reductase and by Synthetic Model Compounds. J. Chem. Phys. B.

[18] Min B., Gao Q., Yan Z., Han X., Hosmer K., Campbell A., Zhu H. (2021). Powering the Remediation of the Nitrogen Cycle: Progress and Perspectives of Electrochemical Nitrate Reduction. Ind. Eng. Chem. Res..

[19] Garcia-Segura S., Lanzarini-Lopes M., Hristovski K., Westerhoff P. (2018). Electrocatalytic reduction of nitrate: Fundamentals to full-scale water treatment applications. Appl. Catal. B Environ..

[20] Dima G.E., de Vooys A.C.A., Koper M.T.M. (2003). Electrocatalytic reduction of nitrate at low concentration on coinage and transition-metal electrodes in acid solutions. J. Electroanal. Chem..

[21] Katsounaros I., Kyriacou G. (2007). Influence of the concentration and the nature of the supporting electrolyte on the electrochemical reduction of nitrate on tin cathode. Electrochim. Acta.

[22] Hristovski K.D., Markovski J. (2017). Engineering metal (hydr)oxide sorbents for removal of arsenate and similar weak-acid oxyanion contaminants: A critical review with emphasis on factors governing sorption processes. Sci. Total Environ..

[23] De Groot M.T., Koper M.T.M. (2004). The influence of nitrate concentration and acidity on the electrocatalytic reduction of nitrate on platinum. J. Electroanal. Chem..

[24] Zhang X., Wang Y., Liu C., Yu Y., Lu S., Zhang B. (2021). Recent advances in non-noble metal electrocatalysts for nitrate reduction. Chem. Eng. J..

[25] Montesinos N., Quici N., Destaillats H., Litter M. (2015). Nitric oxide emission during the reductive heterogeneous photocatalysis of aqueous nitrate with TiO_2_. RSC Adv..

[26] Tugaoen H.O.N., Garcia-Segura S., Hristovski K., Westerhoff P. (2017). Challenges in photocatalytic reduction of nitrate as a water treatment technology. Sci. Total Environ..

[27] Da Cunha M.C.P.M., Weber M., Nart F.C. (1996). On the adsorption and reduction of NO_3_^−^ ions at Au and Pt electrodes studied by in situ FTIR spectroscopy. J. Electroanal. Chem..

[28] De D., Kalu E.E., Tarjan P.P., Englehardt J.D. (2004). Kinetic Studies of the Electrochemical Treatment of Nitrate and Nitrite Ions on Iridium-Modified Carbon Fiber Electrodes. Chem. Eng. Technol..

[29] Goldstein S., Behar D., Rajh T., Rabani J. (2016). Nitrite Reduction to Nitrous Oxide and Ammonia by TiO_2_ Electrons in a Colloid Solution via Consecutive One-Electron Transfer Reactions. J. Phys. Chem. A.

[30] Su J.F., Ruzybayev I., Shah I., Huang C.P. (2016). The electrochemical reduction of nitrate over micro-architectured metal electrodes with stainless steel scaffold. Appl. Catal. B Environ..

[31] De Vooys A.C.A., Beltramo G.L., van Riet B., van Veen J.A.R., Koper M.T.M. (2004). Mechanisms of electrochemical reduction and oxidation of nitric oxide. Electrochim. Acta.

[32] Yoshioka T., Iwase K., Nakanishi S., Hashimoto K., Kamiya K. (2016). Electrocatalytic Reduction of Nitrate to Nitrous Oxide by a Copper-Modified Covalent Triazine Framework. J. Phys. Chem. C.

[33] Zheng J., Lu T., Cotton T.M., Chumanov G. (1999). Photoinduced Electrochemical Reduction of Nitrite at an Electrochemically Roughened Silver Surface. J. Chem. Phys. B.

[34] Yang J., Duca M., Schouten K.J.P., Koper M.T.M. (2011). Formation of volatile products during nitrate reduction on a Sn-modified Pt electrode in acid solution. J. Electroanal. Chem..

[35] Duca M., Cucarella M.O., Rodriguez P., Koper M.T.M. (2010). Direct Reduction of Nitrite to N_2_ on a Pt(100) Electrode in Alkaline Media. J. Am. Chem. Soc..

[36] Duca M., Figueiredo M.C., Climent V., Rodriguez P., Feliu J.M., Koper M.T.M. (2011). Selective Catalytic Reduction at Quasi-Perfect Pt(100) Domains: A Universal Low-Temperature Pathway from Nitrite to N_2_. J. Am. Chem. Soc..

[37] Bartberger M.D., Liu W., Ford E., Miranda K.M., Switzer C., Fukuto J.M., Farmer P.J., Wink D.A., Houk K.N. (2002). The reduction potential of nitric oxide (NO) and its importance to NO biochemistry. Proc. Natl. Acad. Sci. USA.

[38] Dutton A.S., Fukuto J.M., Houk K.N. (2005). Theoretical Reduction Potentials for Nitrogen Oxides from CBS-QB3 Energetics and (C)PCM Solvation Calculations. Inorg. Chem..

[39] Lacasa E., Cañizares P., Llanos J., Rodrigo M.A. (2012). Effect of the cathode material on the removal of nitrates by electrolysis in non-chloride media. J. Hazard. Mater..

[40] Guo S., Heck K., Kasiraju S., Qian H., Zhao Z., Grabow L.C., Miller J.T., Wong M.S. (2018). Insights into Nitrate Reduction over Indium-Decorated Palladium Nanoparticle Catalysts. ACS Catal..

[41] Chaplin B.P., Reinhard M., Schneider W.F., Schüth C., Shapley J.R., Strathmann T.J., Werth C.J. (2012). Critical Review of Pd-Based Catalytic Treatment of Priority Contaminants in Water. Environ. Sci. Technol..

[42] Taguchi S., Feliu J.M. (2007). Electrochemical reduction of nitrate on Pt(S)[n(1 1 1) × (1 1 1)] electrodes in perchloric acid solution. Electrochim. Acta.

[43] Ghodbane O., Sarrazin M., Roué L., Bélanger D. (2008). Electrochemical Reduction of Nitrate on Pyrolytic Graphite-Supported Cu and Pd–Cu Electrocatalysts. J. Electrochem. Soc..

[44] Hu T., Wang C., Wang M., Li C.M., Guo C. (2021). Theoretical Insights into Superior Nitrate Reduction to Ammonia Performance of Copper Catalysts. ACS Catal..

[45] Andersen S.Z., Colic V., Yang S., Schwalbe J.A., Nielander A.C., McEnaney J.M., Enemark-Rasmussen K., Baker J.G., Singh A.R., Rohr B.A. (2019). A rigorous electrochemical ammonia synthesis protocol with quantitative isotope measurements. Nature.

[46] Hodgetts R.Y., Kiryutin A.S., Nichols P., Du H.-L., Bakker J.M., Macfarlane D.R., Simonov A.N. (2020). Refining Universal Procedures for Ammonium Quantification via Rapid ^1^H NMR Analysis for Dinitrogen Reduction Studies. ACS Energy Lett..

[47] Zhao Y., Shi R., Bian X., Zhou C., Zhao Y., Zhang S., Wu F., Waterhouse G.I.N., Wu L.Z., Tung C.H. (2019). Ammonia Detection Methods in Photocatalytic and Electrocatalytic Experiments: How to Improve the Reliability of NH_3_ Production Rates?. Adv. Sci..

[48] Zhou L., Boyd C.E. (2016). Comparison of Nessler, phenate, salicylate and ion selective electrode procedures for determination of total ammonia nitrogen in aquaculture. Aquaculture.

[49] Zhu Y., Yuan D., Lin H., Zhou T.J. (2015). Determination of Ammonium in Seawater by Purge-and-Trap and Flow Injection with Fluorescence Detection. Anal. Lett..

[50] Thomas D.H., Rey M., Jackson P.E. (2002). Determination of inorganic cations and ammonium in environmental waters by ion chromatography with a high-capacity cation-exchange column. J. Chromatogr. A.

[51] Leduy A., Samson R. (1982). Testing of an ammonia ion selective electrode for ammonia nitrogen measurement in the methanogenic sludge. Biotechnol. Lett..

[52] Liu J., Kelley M.S., Wu W., Banerjee A., Douvalis A.P., Wu J., Zhang Y., Schatz G.C., Kanatzidis M.G. (2016). Nitrogenase-mimic iron-containing chalcogels for photochemical reduction of dinitrogen to ammonia. Proc. Natl. Acad. Sci. USA.

[53] Li J., Zhan G., Yang J., Quan F., Mao C., Liu Y., Wang B., Lei F., Li L., Chan A.W.M. (2020). Efficient Ammonia Electrosynthesis from Nitrate on Strained Ruthenium Nanoclusters. J. Am. Chem. Soc..

[54] Han Y., Zhang X., Cai W., Zhao H., Zhang Y., Sun Y., Hu Z., Li S., Lai J., Wang L. (2021). Facet-controlled palladium nanocrystalline for enhanced nitrate reduction towards ammonia. J. Colloid Interface Sci..

[55] Wan H., Bagger A., Rossmeisl J. (2021). Electrochemical Nitric Oxide Reduction on Metal Surfaces. Angew. Chem. Int. Ed..

[56] Long J., Li H., Xiao J. (2023). The progresses in electrochemical reverse artificial nitrogen cycle. Curr. Opin. Electrochem..

[57] Ko B.H., Hasa B., Shin H., Zhao Y., Jiao F. (2022). Electrochemical Reduction of Gaseous Nitrogen Oxides on Transition Metals at Ambient Conditions. J. Am. Chem. Soc..

[58] Liu L., Corma A. (2018). Metal Catalysts for Heterogeneous Catalysis: From Single Atoms to Nanoclusters and Nanoparticles. Chem. Rev..

[59] Fu X., Zhao X., Hu X., He K., Yu Y., Li T., Tu Q., Qian X., Yue Q., Wasielewski M.R. (2020). Alternative route for electrochemical ammonia synthesis by reduction of nitrate on copper nanosheets. Appl. Mater. Today.

[60] Vogler A. (1987). B.C. Gates, L.; Guczi, H. Knözinger (Eds.): Metal Clusters in Catalysis, Vol. 29 aus der Reihe: Studies in Surface Science. Elsevier, Amsterdam, Oxford, New York, Tokyo 1986. 648 Seiten, Preis: Dfl. 195. Ber. Bunsenges. Phys. Chem..

[61] Yang J., Qi H., Li A., Liu X., Yang X., Zhang S., Zhao Q., Jiang Q., Su Y., Zhang L. (2022). Potential-Driven Restructuring of Cu Single Atoms to Nanoparticles for Boosting the Electrochemical Reduction of Nitrate to Ammonia. J. Am. Chem. Soc..

[62] Katsounaros I., Figueiredo M.C., Chen X., Calle-Vallejo F., Koper M.T.M. (2018). Interconversions of nitrogen-containing species on Pt(100) and Pt(111) electrodes in acidic solutions containing nitrate. Electrochim. Acta.

[63] Pérez-Gallent E., Figueiredo M.C., Katsounaros I., Koper M.T.M. (2017). Electrocatalytic reduction of Nitrate on Copper single crystals in acidic and alkaline solutions. Electrochim. Acta.

[64] Chen L.-F., Xie A.-Y., Lou Y.-Y., Tian N., Zhou Z.-Y., Sun S.-G. (2022). Electrochemical synthesis of Tetrahexahedral Cu nanocrystals with high-index facets for efficient nitrate electroreduction. J. Electroanal. Chem..

[65] Hu Q., Qin Y., Wang X., Wang Z., Huang X., Zheng H., Gao K., Yang H., Zhang P., Shao M. (2021). Reaction intermediate-mediated electrocatalyst synthesis favors specified facet and defect exposure for efficient nitrate–ammonia conversion. Energy Environ. Sci..

[66] Xu Y., Wang M., Ren K., Ren T., Liu M., Wang Z., Li X., Wang L., Wang H. (2021). Atomic defects in pothole-rich two-dimensional copper nanoplates triggering enhanced electrocatalytic selective nitrate-to-ammonia transformation. J. Mater. Chem. A.

[67] Sun T., Zhang G., Xu D., Lian X., Li H., Chen W., Su C. (2019). Defect chemistry in 2D materials for electrocatalysis. Mater. Today Energy.

[68] Shi H., Li C., Wang L., Wang W., Meng X. (2022). Selective reduction of nitrate into N_2_ by novel Z-scheme NH_2_-MIL-101(Fe)/BiVO_4_ heterojunction with enhanced photocatalytic activity. J. Hazard. Mater..

[69] Zhao J., Li N., Yu R., Zhao Z., Nan J. (2018). Magnetic field enhanced denitrification in nitrate and ammonia contaminated water under 3D/2D Mn_2_O_3_/g-C_3_N_4_ photocatalysis. Chem. Eng. J..

[70] Adamu H., McCue A.J., Taylor R.S.F., Manyar H.G., Anderson J.A. (2017). Simultaneous photocatalytic removal of nitrate and oxalic acid over Cu_2_O/TiO_2_ and Cu_2_O/TiO_2_-AC composites. Appl. Catal. B Environ..

[71] Kumar A., Lee J., Kim M.G., Debnath B., Liu X., Hwang Y., Wang Y., Shao X., Jadhav A.R., Liu Y. (2022). Efficient Nitrate Conversion to Ammonia on f-Block Single-Atom/Metal Oxide Heterostructure via Local Electron-Deficiency Modulation. ACS Nano.

[72] Yu T., Liu L., Yang F. (2017). Heterojunction between anodic TiO_2_/g-C_3_N_4_ and cathodic WO3/W nano-catalysts for coupled pollutant removal in a self-biased system. Chin. J. Catal..

[73] Deng Z., Ma C., Fan X., Li Z., Luo Y., Sun S., Zheng D., Liu Q., Du J., Lu Q. (2022). Construction of CoP/TiO_2_ nanoarray for enhanced electrochemical nitrate reduction to ammonia. Mater. Today Phys..

[74] Liu S., Cui L., Yin S., Ren H., Wang Z., Xu Y., Li X., Wang L., Wang H. (2022). Heterointerface-triggered electronic structure reformation: Pd/CuO nano-olives motivate nitrite electroreduction to ammonia. Appl. Catal. B Environ..

[75] Wang Y., Zhou W., Jia R., Yu Y., Zhang B. (2020). Unveiling the Activity Origin of a Copper-based Electrocatalyst for Selective Nitrate Reduction to Ammonia. Angew. Chem. Int. Ed..

[76] Chen G.-F., Yuan Y., Jiang H., Ren S.-Y., Ding L.-X., Ma L., Wu T., Lu J., Wang H. (2020). Electrochemical reduction of nitrate to ammonia via direct eight-electron transfer using a copper–molecular solid catalyst. Nat. Energy.

[77] Reyter D., Reyter D., Chamoulaud G., Chamoulaud G., Bélanger D., Roué L. (2006). Electrocatalytic reduction of nitrate on copper electrodes prepared by high-energy ball milling. J. Electroanal. Chem..

[78] Zhao Y., Liu Y., Zhang Z., Mo Z., Wang C., Gao S. (2022). Flower-like open-structured polycrystalline copper with synergistic multi-crystal plane for efficient electrocatalytic reduction of nitrate to ammonia. Nano Energy.

[79] Yuan J., Xing Z., Tang Y., Liu C. (2021). Tuning the Oxidation State of Cu Electrodes for Selective Electrosynthesis of Ammonia from Nitrate. ACS Appl. Mater. Interfaces.

[80] Zhao X., Jia X., He Y., Zhang H., Zhou X., Zhang H., Zhang S., Dong Y., Hu X., Kuklin A.V. (2021). Two-dimensional BCN matrix inlaid with single-atom-Cu driven electrochemical nitrate reduction reaction to achieve sustainable industrial-grade production of ammonia. Appl. Mater. Today.

[81] Qin J., Chen L., Wu K., Wang X., Zhao Q., Li L., Liu B., Ye Z. (2022). Electrochemical Synthesis of Ammonium from Nitrates via Surface Engineering in Cu_2_O(100) Facets. ACS Appl. Energy Mater..

[82] Wang Y., Xu A., Wang Z., Huang L., Li J., Li F., Wicks J., Luo M., Nam D.H., Tan C.S. (2020). Enhanced Nitrate-to-Ammonia Activity on Copper-Nickel Alloys via Tuning of Intermediate Adsorption. J. Am. Chem. Soc..

[83] Ge Z.-X., Wang T.-J., Ding Y., Yin S.-B., Li F.-M., Chen P., Chen Y. (2022). Interfacial Engineering Enhances the Electroactivity of Frame-Like Concave RhCu Bimetallic Nanocubes for Nitrate Reduction. Adv. Energy Mater..

[84] Liu J.-X., Richards D., Singh N., Goldsmith B.R. (2019). Activity and Selectivity Trends in Electrocatalytic Nitrate Reduction on Transition Metals. ACS Catal..

[85] Barrabés N., Just J., Dafinov A., Medina F., Fierro J.L.G., Sueiras J.E., Salagre P., Cesteros Y. (2006). Catalytic reduction of nitrate on Pt-Cu and Pd-Cu on active carbon using continuous reactor: The effect of copper nanoparticles. Appl. Catal. B Environ..

[86] Zhang Q., Ding L., Cui H., Zhai J., Wei Z., Li Q. (2014). Electrodeposition of Cu-Pd alloys onto electrophoretic deposited carbon nanotubes for nitrate electroreduction. Appl. Surf. Sci..

[87] Lei X., Liu F., Li M., Ma X., Wang X., Zhang H. (2018). Fabrication and characterization of a Cu-Pd-TNPs polymetallic nanoelectrode for electrochemically removing nitrate from groundwater. Chemosphere.

[88] Shih Y.-J., Wu Z.-L., Lin C.-Y., Huang Y.-H., Huang C.-P. (2020). Manipulating the crystalline morphology and facet orientation of copper and copper-palladium nanocatalysts supported on stainless steel mesh with the aid of cationic surfactant to improve the electrochemical reduction of nitrate and N_2_ selectivity. Appl. Catal. B Environ..

[89] Kerkeni S., Lamy-Pitara E., Barbier J. (2002). Copper–platinum catalysts prepared and characterized by electrochemical methods for the reduction of nitrate and nitrite. Catal. Today.

[90] Wang J., Teng W., Ling L., Fan J., Zhang W.x., Deng Z.-l. (2020). Nanodenitrification with bimetallic nanoparticles confined in N-doped mesoporous carbon. Environ. Sci. Nano.

[91] Liu H., Lang X., Zhu C., Timoshenko J., Rüscher M., Bai L., Guijarro N., Yin H., Peng Y., Li J. (2022). Efficient Electrochemical Nitrate Reduction to Ammonia with Copper-Supported Rhodium Cluster and Single-Atom Catalysts. Angew. Chem. Int. Ed..

[92] He W., Zhang J., Dieckhofer S., Varhade S., Brix A.C., Lielpetere A., Seisel S., Junqueira J.R.C., Schuhmann W. (2022). Splicing the active phases of copper/cobalt-based catalysts achieves high-rate tandem electroreduction of nitrate to ammonia. Nat. Commun..

[93] Mattarozzi L., Cattarin S., Comisso N., Gambirasi A., Guerriero P., Musiani M., Vázquez-Gómez L., Verlato E. (2014). Hydrogen evolution assisted electrodeposition of porous Cu-Ni alloy electrodes and their use for nitrate reduction in alkali. Electrochim. Acta.

[94] Mattarozzi L., Cattarin S., Comisso N., Gerbasi R., Guerriero P., Musiani M., Vázquez-Gómez L., Verlato E. (2015). Electrodeposition of Compact and Porous Cu-Zn Alloy Electrodes and Their Use in the Cathodic Reduction of Nitrate. J. Electrochem. Soc..

[95] Zhang Y., Chen X., Wang W., Yin L., Crittenden J.C. (2022). Electrocatalytic nitrate reduction to ammonia on defective Au_1_Cu (111) single-atom alloys. Appl. Catal. B Environ..

[96] Xu Y., Ren K., Ren T., Wang M., Liu M., Wang Z., Li X., Wang L., Wang H. (2021). Cooperativity of Cu and Pd active sites in CuPd aerogels enhances nitrate electroreduction to ammonia. Chem. Commun..

[97] Yin H., Chen Z., Xiong S., Chen J., Wang C., Wang R., Kuwahara Y., Luo J., Yamashita H., Peng Y. (2021). Alloying effect-induced electron polarization drives nitrate electroreduction to ammonia. Chem. Catal..

[98] Fang J.-Y., Zheng Q.-Z., Lou Y.-Y., Zhao K.-M., Hu S.-N., Li G., Akdim O., Huang X.-Y., Sun S.-G. (2022). Ampere-level current density ammonia electrochemical synthesis using CuCo nanosheets simulating nitrite reductase bifunctional nature. Nat. Commun..

[99] Butcher D.P., Gewirth A.A. (2016). Nitrate reduction pathways on Cu single crystal surfaces: Effect of oxide and Cl^−^. Nano Energy.

[100] Bae S.-E., Stewart K.L., Gewirth A.A. (2007). Nitrate Adsorption and Reduction on Cu(100) in Acidic Solution. J. Am. Chem. Soc..

[101] Magnussen O.M. (2002). Ordered Anion Adlayers on Metal Electrode Surfaces. Chem. Rev..

[102] Kleinert M., Cuesta A., Kibler L.A., Kolb D.M. (1999). In-situ observation of an ordered sulfate adlayer on Au(100) electrodes. Surf. Sci..

[103] Li W., Xiao C., Zhao Y., Zhao Q., Fan R., Xue J. (2016). Electrochemical Reduction of High-Concentrated Nitrate Using Ti/TiO_2_ Nanotube Array Anode and Fe Cathode in Dual-Chamber Cell. Catal. Lett..

[104] Zhang C., He D., Ma J., Waite T.D. (2018). Active chlorine mediated ammonia oxidation revisited: Reaction mechanism, kinetic modelling and implications. Water Res..

